# Autism Diagnostic Interview-Revised (ADI-R) Algorithms for Toddlers and Young Preschoolers: Application in a Non-US Sample of 1,104 Children

**DOI:** 10.1007/s10803-015-2372-2

**Published:** 2015-02-15

**Authors:** Annelies de Bildt, Sjoerd Sytema, Eric Zander, Sven Bölte, Harald Sturm, Nurit Yirmiya, Maya Yaari, Tony Charman, Erica Salomone, Ann LeCouteur, Jonathan Green, Ricardo Canal Bedia, Patricia García Primo, Emma van Daalen, Maretha V. de Jonge, Emilía Guðmundsdóttir, Sigurrós Jóhannsdóttir, Marija Raleva, Meri Boskovska, Bernadette Rogé, Sophie Baduel, Irma Moilanen, Anneli Yliherva, Jan Buitelaar, Iris J. Oosterling

**Affiliations:** 1 Department of Psychiatry, University Medical Center Groningen, University of Groningen, Groningen, The Netherlands; 2 Accare University Center for Child and Adolescent Psychiatry, PO Box 660, 9700 AR Groningen, The Netherlands; 3 Center of Neurodevelopmental Disorders (KIND), Department of Women’s and Children’s Health, Karolinska Institutet, Stockholm, Sweden; 4 Division of Child and Adolescent Psychiatry, Stockholm County Council, Stockholm, Sweden; 5 Department of Psychology, The Hebrew University of Jerusalem, Jerusalem, Israel; 6 Department of Psychology, Institute of Psychiatry, Psychology and Neuroscience, King’s College, London, UK; 7 Institute of Health and Society, Newcastle University, Newcastle, UK; 8 Psychiatry Research Group, University of Manchester, Manchester, UK; 9 INICO, University of Salamanca, Salamanca, Spain; 10 Rare Disease Research Institute, National Institute of Health, Barcelona, Spain; 11 Department of Child and Adolescent Psychiatry, University Medical Center Utrecht, Utrecht, The Netherlands; 12 The State Diagnostic and Counseling Center, Kópavogur, Iceland; 13 Department of Child and Adolescent Psychiatry, University Clinic of Psychiatry, Skopje, Republic of Macedonia; 14 Octogone/CERPP, Toulouse University, Toulouse, France; 15 Faculty of Humanities, Logopedics, University of Oulu, Oulu, Finland; 16 Karakter Child and Adolescent Psychiatry University Center, Nijmegen, The Netherlands; 17 Department of Cognitive Neuroscience, Donders Institute for Brain, Cognition and Behaviour, Radboud University Nijmegen Medical Center, Nijmegen, The Netherlands; 18 Department of Psychiatry, Nijmegen Center for Evidence-Based Practice (NCEBP), Radboud University Nijmegen Medical Center, Nijmegen, The Netherlands; 19 Clinic of Child Psychiatry, University Hospital of Oulu, Oulu, Finland

**Keywords:** Autism spectrum disorders, Early diagnosis, Assessment

## Abstract

The current study aimed to investigate the Autism Diagnostic Interview-Revised (ADI-R) algorithms for toddlers and young preschoolers (Kim and Lord, J Autism Dev Disord 42(1):82–93, 2012) in a non-US sample from ten sites in nine countries (n = 1,104). The construct validity indicated a good fit of the algorithms. The diagnostic validity was lower, with satisfactorily high specificities but moderate sensitivities. Young children with clinical ASD and lower language ability were largely in the mild-to-moderate or moderate-to-severe concern ranges of the ADI-R, nearly half of the older and phrase speech ASD-group fell into the little-to-no concern range. Although broadly the findings support the toddler algorithms, further work is required to understand why they might have different properties in different samples to further inform research and clinical use.

## Introduction

Parallel to the increased evidence for the effectiveness of early intervention for children with autism spectrum disorders (ASDs; Oono et al. 2013), great improvements have been accomplished in early recognition and diagnosis of ASD (Al-Qabandi et al. 2011; Oosterling et al. 2010c; Yirmiya and Charman 2010; Zwaigenbaum et al. 2013). Diagnostic instruments such as the Autism Diagnostic Observation Schedule (ADOS; Lord et al. 1999) and later ADOS-Second Edition (ADOS-2; Lord et al. 2012a, b) and the Autism Diagnostic Interview-Revised (ADI-R; Rutter et al. 2003b) have evolved over the years. They now provide valuable information for clinicians in order to establish an early diagnosis (Charman and Gotham 2013). For the ADOS-2, the revised algorithms for modules 1 and 2 and the algorithms for the Toddler module have shown significant value in the early diagnosis of ASD (Gotham et al. 2007, 2008; Luyster et al. 2009; Molloy et al. 2011; Oosterling et al. 2010b, Overton et al. 2008).

The ADI-R is known to be reliable in older children (Lord et al. 1994). However, for younger children the existing algorithm was not optimal (e.g. Lord et al. 1993; Oosterling et al. 2010a; Risi et al. 2006; Ventola et al. 2006; Wiggins and Robins 2008). Recently, Kim and Lord (2012) proposed new algorithms for toddlers and preschool children, aged 12–47 months, aiming to improve ADI-R validity for these young children. The algorithms were developed in a large US dataset (Michigan sample, N = 829) of toddlers and preschoolers aged 12–47 months, with a nonverbal mental age from 10 months and higher. The algorithms for toddlers and preschoolers have been constructed in line with the revised algorithms of the ADOS-2 (Gotham et al. 2007) and the DSM-5 ASD criteria (APA 2013). They are more specific, since the algorithms contain slightly different items for different developmental groups based on age and language level. These developmental groups, referred to as developmental ‘cells’, are defined as (a) all children 12–20 months of age as well as nonverbal children 21–47 months of age (12-20/NV21-47), (b) all children 21–47 months with single words (SW21-47), and (c) all children 21–47 months with phrase speech (PH21-47).

The new algorithms are shorter (13–20 items) than the original algorithms (33–39 items) and contain three domains. The algorithms are based on items from the standard ADI-R version (Rutter et al. 2003b) that also appear in the toddler version (Kim and Lord 2012). The first domain is the Social Affect Domain (SA; cells 12-20/NV21-47 and SW21-47) or the Social Communication Domain (SC; cell PH21-47), which contains items on social interaction and communication. The second domain is the Restrictive, Repetitive Behavior domain (RRB). The third domain is either Imitation, Gesture and Play (IGP; 12-20/NV21-47 and SW21-47) or Reciprocal and Peer Interaction (RPI; cell PH21-47). For the 12-20/NV21-47 and the SW21-47 cells algorithm cutoffs are based on two domains, namely SA and RRB. The IGP domain was not included in the algorithm in these cells, because it did not discriminate ASD from non-spectrum diagnoses or typically developing children when age, IQ and the other domains were included in the analyses. For the PH21-47 cell cutoffs are based on all three domains.

For each developmental cell two cutoffs for ASD versus non-ASD have been defined: one for research (higher threshold, more restrictive; higher specificity, lower sensitivity) and one for clinical use (lower threshold, more inclusive; higher sensitivity, lower specificity). Additional to the classification based on these cutoffs, ranges of concern have been provided, reflecting little-to-no, mild-to-moderate, or moderate-to-severe concern, in order to represent the severity of autism symptoms.

The algorithm development study of Kim and Lord (2012) in the Michigan sample indicated a good fit of the three factor structure in all three developmental cells. It also showed improved diagnostic validity of the ADI-R classification based on the clinical and research cutoffs with a best estimate clinical ASD diagnosis as the criterion, compared to the original algorithm. The ADI-R classifications showed high sensitivities (ADI-R clinical cutoff .80–.94, research cutoff .80–.84) and specificities (ADI-R clinical cutoff .70–.81, research cutoff .82–.90). Correlations between scores on the algorithms and age and level of functioning indicated relative independence of these characteristics.

The authors have replicated the algorithms in two independent US samples (both described in Kim et al. 2013). One study had a relatively large (CPEA/STAART, N = 641), the other a smaller sample size (NIMH, N = 167). In both studies, the three factor structure was well replicated. The specific developmental cells were found to be applicable and correlations between participant characteristics and algorithm scores remained low. Additionally, the improved diagnostic validity of the ADI-R toddler algorithms was confirmed. Within the CPEA/STAART sample, sensitivities were comparable to the Michigan sample, and specificities were noticeably improved. Within the NIMH sample sensitivities were higher compared to the Michigan sample, and specificities were comparable, except for the 12-20/NV21-47 group which showed a slightly lower specificity. Of note is that in all US samples, differentiating children with ASD from those referred for ASD but who received non-spectrum diagnoses, was difficult.

Logistic regressions in the CPEA/STAART sample confirmed an independent contribution of the SA and SC domains to the ASD classification based on the ADI-R toddler algorithms in all developmental cells. Independent contribution of RRB varied over the developmental cells: for older and more able children, the RRB items contributed significantly to an ASD classification whereas for younger and/or more impaired children this was less the case.

In line with the Michigan sample, over 80 % of the children with ASD in the CPEA/STAART sample fell into the two ranges of clinical concern. The percentage of NS cases in the risk ranges was lower than in the Michigan sample. Because of limited sample sizes within cells, logistic regressions and ranges of concern have not been examined for the NIMH sample.

Application of the ADI-R toddler algorithms in other samples across sites, with independent, well-defined populations with and without ASD is important in investigating the generalizability of the ADI-R toddler algorithms (Kim and Lord 2012).

The current paper aims to make a modest contribution to examining aspects of the validity of the ADI-R algorithms for toddlers and preschoolers as proposed by Kim and Lord (2012): the factor structure and sensitivity and specificity. This is attempted in a large, fully independent, varied, non-US sample (N = 1,104). In addition, use of clinical and research cutoffs as well as ranges of concern were evaluated. This study was initiated and realized within the European network: ESSEA (Enhancing the Scientific Study of Early Autism) COST action (European Cooperation in Science and Technology). This network strives to establish an interdisciplinary scientific network to advance the pace of discovery on the earliest signs of autism (Bölte et al. 2013); to combine techniques from cognitive neuroscience with those from the clinical sciences; and to generate European practice guidelines on early identification and intervention (for more information, see: www.cost-essea.com).

## Methods

### Participants

All sites of the COST-ESSEA network (consisting of 80 scientists in 23 countries) were invited to contribute existing databases to participate in the current study. Ten sites that had relevant data to contribute participated, resulting in the collection of 1,187 cases outside the US. To be included in the current sample, the toddlers and young preschoolers had to be between 12 months and 47 months 30 days old with nonverbal mental ages from 10 months and higher, had to have an ADI-R available with scores on all domains as specified for developmental cell and had to have received a best clinical estimate diagnosis (BCE), resulting in an N of 1,104. Additionally, research reliability of administration and scoring of the ADI-R was required.

The sample (74.0 % males) had a mean age of 34.6 months (SD = 8.06). Just over half of the children (56.1 %) had a BCE ASD diagnosis. Due to the young age of the sample and in line with DSM-5, no differentiation was made between autistic disorder (AD) and non-autism ASD (formerly, pervasive developmental disorder). Another 24.5 % had a non-spectrum diagnosis (NS) and 19.5 % were typically developing (TD). The 12-20/NV21-47 cell mainly consisted of children with ASD (N = 263) with 60 children with NS disorders and only seven TD children, included for determining sensitivity and specificity. The SW21-47 cell contained 192 cases with ASD, 90 with NS disorders and 42 TD. In the PH21-47 cell, 36.4 % had an ASD (N = 164), with almost equally many with NS disorders (120) and TD (166). The non-spectrum diagnoses were classified following Kim and Lord (2012) as: language delay (N = 112), nonspecific intellectual disability (N = 39), Attention Deficit/Hyperactivity Disorder (ADHD; N = 34), nonspecific developmental delay (N = 28), anxiety or internalizing emotion regulation problems (N = 27), externalizing emotion regulation problems (N = 10), attachment (N = 2) and other (N = 18). In Table 1, the participant characteristics are presented for the total sample.

**Table 1:** Participant characteristics

Clinical classification	12-20/NV21-47			SW21-47			PH21-47		
	N	Mean	SD	N	Mean	SD	N	Mean	SD
*ASD*									
Age (months)	263	32.2^a,b^	7.4	192	36.1^a,c^	6.5	164	40.2^c^	4.8
NVIQ	230	57.7^d,e^	19.0	164	69.4^d,e^	19.0	136	81.9^d,e^	19.0
ADI SA/SC	263	11.6^c,f^	3.9	192	9.8^c,f^	4.2	164	8.6^c,f^	4.6
ADI RRB	263	3.3^f^	2.5	192	3.5^c,f^	3.0	164	2.9^c,f^	2.5
ADI IGP/RPI	263	10.4^c,f^	2.3	192	6.7^c,f^	2.5	164	2.8^c,f^	1.6
ADI tot2dom	263	14.9	5.5	192	13.3	6.2	164	11.5	6.0
ADI tot3dom	263	25.2	7.1	192	20.0	8.0	164	14.3	7.1
ADOS T NW SA	–			–			–		
ADOS T NW RRB	–			–			–		
ADOS T SW SA	1	22.0		1	6.0		–		
ADOS T SW RRB	1	5.0		1	14.0		–		
ADOS 1 NW SA	135	14.8^f^	3.3	62	12.5	3.1	13	11.2^a^	3.1
ADOS 1 NW RRB	135	3.4^f^	1.9	62	2.4	1.8	13	1.8^c,f^	1.6
ADOS 1 SW SA	60	16.4	3.1	98	12.6^c,f^	4.5	75	10.4^c,f^	5.0
ADOS 1 SW RRB	60	3.8	1.6	98	2.4^f^	1.7	75	2.5^c,f^	1.8
ADOS 2 SA	–			3	14.7	4.2	59	10.5^c,f^	4.5
ADOS 2 RRB	–			3	4.7	1.5	59	2.7^c,f^	2.3
*Non*-*spectrum disorders*									
Age (months)	60	25.6^a^	9.3	90	33.4^a,g^	7.7	120	38.1^h^	6.0
NVIQ	44	74.0^d,i^	25.1	81	82.6^d,j^	18.8	116	93.1^d,j^	16.7
ADI SA/SC	60	3.6^f^	3.1	90	4.8^f,g^	3.6	120	4.2^f,g^	3.8
ADI RRB	60	.9^f^	1.5	90	1.3^f^	1.9	120	1.4^f,h^	1.9
ADI IGP/RPI	60	4.5^f^	3.6	90	3.7^f,g^	2.5	120	2.1^f,g^	1.6
ADI tot2dom	60	4.5	4.0	90	6.1	4.4	120	5.6	4.9
ADI tot3dom	60	8.9	6.9	90	9.8	6.1	120	7.7	5.9
ADOS T NW SA	–			4	7.3	6.9	–		
ADOS T NW RRB	–			4	2.3	1.3	–		
ADOS T SW SA	2	5.0	7.1	2	4.0	1.4	3	5.0	6.2
ADOS T SW RRB	2	.5	.7	2	.5	.7	3	2.7	.6
ADOS 1 NW SA	34	5.1^f^	5.0	18	7.4	4.9	2	4.5^a^	.7
ADOS 1 NW RRB	34	1.0^f^	1.4	18	1.2	1.4	2	1.0^f,h^	1.4
ADOS 1 SW SA	8	2.9	1.7	41	4.5^f^	3.8	62	3.8^f^	3.0
ADOS 1 SW RRB	8	.6	.7	41	1.0^f^	1.2	62	.6^f^	.8
ADOS 2 SA				6	2.7	4.6	39	5.4^f,g^	3.2
ADOS 2 RRB				6	.7	.8	39	1.2^f,h^	1.4
*Typical development*									
Age (months)	7	22.3^b^	7.3	42	26.4^c,g^	5.3	166	35.1^d,h^	8.5
NVIQ	7	97.7^e,i^	20.4	41	101.4^e,j^	12.3	164	109.3^e,j^	13.2
ADI SA/SC	7	4.1^c^	5.3	42	1.8^c,g^	3.0	166	1.0^c,g^	1.8
ADI RRB	7	1.6	2.1	42	1.0^c^	1.8	166	.7^c,h^	1.2
ADI IGP/RPI	7	4.4^c^	4.5	42	1.6^c,g^	1.8	166	.6^c,g^	.9
ADI tot2dom	7	5.7	7.4	42	2.8	4.3	166	1.7	2.5
ADI tot3dom	7	10.1	11.8	42	4.5	5.6	166	2.3	3.0
ADOS T NW SA	2	1.5	2.1	15	2.5	3.0	24	1.5	1.8
ADOS T NW RRB	2	.5	.7	15	.9	1.2	24	.5	.7
ADOS T SW SA	2	1.5	2.1	18	1.4	1.3	36	1.4	1.7
ADOS T SW RRB	2	0	0	18	.8	1.4	36	.5	.8
ADOS 1 NW SA	2	10.0	7.1	–			2	12.0^c,h^	0
ADOS 1 NW RRB	2	4.5	3.5	–			2	2.0	2.8
ADOS 1 SW SA	1	1.0		7	5.3^c^	5.2	33	1.7^c^	2.0
ADOS 1 SW RRB	1	0		7	1.3	2.1	33	.6^c^	.9
ADOS 2 SA	–			–			66	1.3^c,g^	1.5
ADOS 2 RRB	–			–			66	.4^c,h^	.7

*12*-*20/NV21*-*47* all children from 12 through 20 months and nonverbal children from 21 through 47 months; *ADItot2dom* total score on SA/SC + RRB domains combined; *ADItot3dom* total score on SA/SC + RRB + IGP/RPI domains combined; *IGP* Imitation, Gestures and Play Total for the 12-20/NV21-47 and SW21-47 algorithms; *PH21*-*47* Children from 21 through 47 months with phrase speech; *RPI* Reciprocal and peer Interaction total for the PH21-47 algorithm; *RRB* Restricted and repetitive Behaviors Total; *SA* Social affect Total for the 12-20/NV21-47 and SW21-47 algorithms *SC* Social Communication Total for the PH21-47 algorithm; *SW21*-*47* Children from 21 through 47 months with single words

Significant differences with Bonferroni correction

^a^ASD > NS *p* < .05

^b^ASD > TD *p* < .05

^c^ASD > TD *p* < .001

^d^NS > ASD *p* < .001

^e^TD > ASD *p* < .001

^f^ASD > NS *p* < .001

^g^NS > TD *p* < .001

^h^NS > TD *p* < .05

^i^TD > NS *p* < .01

^j^TD > NS *p* < .001

Since the ESSEA network was formed in order to generate European practice guidelines on early identification and intervention (amongst other things), existing datasets from the participating sites did not fully match in character, background and diagnostic procedure. The total sample thus consists of children from various settings and characteristics per site are presented in Table 2. The 10 participating sites are:

**Table 2:** Participant characteristics per site

	Sweden	NL Nijmegen	Israel	UK	Spain	NL Utrecht	Iceland	Macedonia	France	Finland
N	234	230	206	188	115	42	39	20	17	13
*Sex*										
Male %	73.9	81.3	51.9	86.7	80.0	69.0	76.9	85.0	76.5	46.2
*Age in months*										
Mean (SD)	36.9 (6.9)	32.9 (5.6)	32.6 (8.8)	35.7 (9.5)	32.6 (6.4)	39.6 (7.2)	39.0 (6.1)	41.5 (5.2)	34.8 (6.5)	14.85 (1.2)
*IQ*										
NVIQ mean (SD)	81.1 (18.1)	77.2 (24.0)	106.5 (14.9)	69.8 (20.7)	60.5 (21.2)	113.0 (16.6)	91.7 (12.6)	40.4 (10.6)	90.0 (23.9)	
*Developmental cell*										
12-20/NV21-47 %	29.5	24.8	2.4	49.5	53.9	21.4	15.4	75.0	5.9	100.0
SW21-47 %	35.0	33.0	20.4	28.2	26.1	26.2	48.7	25.0	35.3	0
PH21-47 %	35.5	42.2	77.2	22.3	20.0	52.4	35.9	0	58.8	0
*BE Clinical Diagnosis*										
ASD %	62.4	67.0	3.4	78.2	61.7	76.2	82.1	100.0	58.8	0
NS %	32.1	32.6	8.3	16.0	35.7	19.0	17.9	0	23.5	100.0
TD %	5.6	.4	88.3	5.9	2.6	4.8	0	0	17.6	0
*ADI*-*R scores*										
Above clin cutoff %	46.6	46.5	2.9	75.5	53.9	69.0	64.1	100.0	70.6	0
Above res cutoff %	29.9	25.7	1.5	68.6	43.5	54.8	33.3	100.0	58.8	0
*12*-*20/NV21*-*47*										
*ASD*										
SA mean (SD*)	9.2 (3.7)	10.2 (3.7)	10	13.1 (2.9)	12.0 (3.7)	12.6 (5.1)	13.0 (3.5)	16.1 (2.2)	17	
RRB mean (SD*)	3.2 (2.2)	1.7 (2.4)	2	4.7 (2.4)	2.3 (1.9)	3.6 (1.4)	3.6 (1.1)	4.9 (2.3)	3	
IGP mean (SD*)	8.8 (2.8)	9.7 (2.5)	11	11.4 (.9)	10.8 (2.0)	10.0 (2.2)	11.8 (.4)	11.1 (1.3)	12	
*NS*										
SA mean (SD*)	4.3 (3.1)	4.4 (3.0)	0	4.4 (3.0)	3.9 (2.7)	4	12			.9 (1.5)
RRB mean (SD*)	1.7 (1.7)	.6 (.9)	0	1.2 (1.9)	.6 (1.1)	5	1			.1 (.3)
IGP mean (SD*)	3.8 (3.2)	7.4 (3.7)	0	5.3 (3.8)	4.6 (3.2)	12	9			2.2 (2.0)
*TD*										
SA mean (SD*)	8.5 (.7)		0 (0)	12	0					
RRB mean (SD*)	3.0 (1.4)		0 (0)	5	0					
IGP mean (SD*)	8.0 (.0)		.3 (.6)	11	3					
*SW21*-*47*										
SA mean (SD*)	8.2 (3.5)	8.8 (4.2)	8	11.7 (3.7)	11.5 (4.1)	10.9 (4.8)	9.2 (3.9)	17.4 (1.7)	11.0 (1.0)	
RRB mean (SD*)	2.9 (2.4)	1.5 (2.4)	3	6.0 (2.7)	3.1 (2.7)	4.7 (2.6)	2.5 (2.0)	8.2 (3.3)	6.0 (1.0)	
IGP mean (SD*)	5.2 (2.6)	6.8 (2.6)	7	7.7 (2.1)	8.6 (1.5)	6.7 (2.2)	6.5 (2.0)	9.6 (.9)	6.3 (.6)	
*NS*										
SA mean (SD*)	5.6 (3.5)	5.0 (3.6)	2.0 (2.3)	4.3 (2.3)	3.7 (3.0)	12.5 (4.9)	4.7 (4.5)		.5 (.7)	
RRB mean (SD*)	1.7 (1.5)	.4 (.8)	.3 (.5)	2.0 (3.2)	.9 (1.8)	4.0 (2.8)	1.8 (2.3)		3.5 (4.9)	
IGP mean (SD*)	3.4 (2.4)	4.5 (2.3)	1.3 (1.5)	5.0 (3.3)	2.9 (1.9)	8.5 (2.1)	3.3 (2.3)		5.0 (.0)	
*TD*										
SA mean (SD*)	9	2	1.0 (1.4)	8.0 (7.0)	6				0	
RRB mean (SD*)	0	0	.8 (1.1)	5.0 (4.4)	0				0	
IGP mean (SD*)	5	0	1.3 (1.5)	4.0 (1.0)	6				1	
*PH21*-*47*										
SC mean (SD*)	7.5 (4.8)	7.9 (3.9)	9.2 (5.0)	9.5 (5.5)	12.1 (5.0)	9.2 (4.8)	7.4 (2.6)	12.5 (3.9)		
RRB mean (SD*)	3.0 (2.6)	1.7 (1.8)	3.0 (3.2)	4.6 (3.2)	2.5 (1.6)	3.3 (1.7)	2.6 (1.4)		4.3 (2.1)	
RPI mean (SD*)	3.1 (1.1)	2.6 (1.8)	3.2 (1.6)	2.8 (2.0)	3.5 (1.8)	2.1 (1.6)	1.9 (.9)		3.5 (1.0)	
*NS*										
SC mean (SD*)	4.2 (3.3)	4.9 (4.0)	.7 (1.1)	4.4 (5.5)	2.5 (1.8)	6.2 (3.1)			9.5 (10.6)	
RRB mean (SD*)	1.7 (2.0)	.9 (1.2)	1.0 (1.5)	2.0 (2.8)	1.1 (1.4)	4.6 (3.4)			3.0 (2.8)	
RPI mean (SD*)	2.5 (1.2)	2.2 (1.7)	.9 (1.1)	1.1 (1.9)	1.4 (1.6)	2.4 (2.1)			2.5 (.7)	
*TD*										
SC mean (SD*)	4.2 (3.2)		.6 (1.1)	3.6 (2.5)	0	5.0 (.0)			.5 (.7)	
RRB mean (SD*)	1.8 (1.5)		.6 (1.0)	1.7 (2.9)	0	1.5 (.7)			.0 (.0)	
RPI mean (SD*)	1.9 (1.6)		.5 (.8)	1.0 (1.0)	0	1.5 (2.1)			.0 (.0)	

*12*-*20/NV21*-*47* all children from 12 through 20 months and nonverbal children from 21 through 47 months, *ADI*-*R scores*  *% above clinical cutoff*The percentage of children with a ADI-R score above the clinical cutoff, *ADI*-*R scores*  *% above research cutoff* The percentage of children with a ADI-R score above the research cutoff, *ASD* children with ASD diagnosis, *IGP* Imitation, Gestures and Play Total for the 12-20/NV21-47 and SW21-47 algorithm, *NS* children with non-spectrum diagnoses, *PH21*-*47* Children from 21 through 47 months with phrase speech, *RPI* Reciprocal and peer Interaction total for the PH21-47 algorithm, *RRB* Restricted and repetitive Behaviors Total, *SA* Social affect Total for the 12-20/NV21-47 and SW21-47 algorithms, *SC* Social Communication Total for the PH21-47 algorithm, *SW21*-*47* Children from 21 through 47 months with single words, *TD* typically developing children

* When no SD is presented, N = 1in the specific cell

1. *Sweden*, the Neuropsychiatric Resource Team Southeast, Division of Child and Adolescent Psychiatry, Stockholm County Council; N = 234 (see Zander et al. 2014),
2. The Netherlands *Nijmegen*, University Center for Child and Adolescent Psychiatry, N = 230 (see Oosterling et al. 2010c),
3. The Netherlands *Utrecht*, University Center for Child and Adolescent Psychiatry, N = 42,
4. *Israel*, the Hebrew University of Jerusalem, N = 206,
5. *United Kingdom*, Preschool Autism Communication Trial study (PACT; Green et al. 2010) N = 92; CHAT screening study (Baird et al. 2000) N = 27; CHAT intervention study (Drew et al. 2002) N = 26; PPP study (unpublished data) N = 43); total N = 188,
6. *Spain,* the Salamanca University ASD Unit, N = 115 (see Canal-Bedia et al. 2011),
7. *Iceland,* the State Diagnostic and Counseling Center, N = 39,
8. *Macedonia,* the University Clinic of Psychiatry, N = 20,
9. *France,* the University of Toulouse and CeRESA, an organization for diagnosis and intervention for ASD, N = 17, and
10. *Finland*, Oulu University Clinic of Child Psychiatry, N = 13.

### Measures and Procedures

#### Enrollment and Site Differences

In Table 3, the procedures of enrollment and the diagnostic procedures are presented for all sites.

**Table 3:** Diagnostic procedures per site

	Sweden	NL Nijmegen	Israel	UK	Spain	NL Utrecht	Iceland	Macedonia	France	Finland
Total n	234	230	206	188	115	42	39	20	17	13
*Recruitment*										
Clinical referral (reason)	234 (dev pr)					42 (ASD/dev pr)	39 (ASD)	20 (dev pr)	17 (ASD)	
Screening (population)		230 (HR)^1^		96 (GP)	115 (HR)					13 (GP)
General population			206							
Research referral				92						
*Diagnostic team*										
Pediatrician	X	–	–	X	X	–	X	X	–	–
(Child) psychiatrist	X	X	–	X	X	X	–	X	X	–
(Child) psychologist	X	X	X	–	X	X	X	X	X	X
Social worker	X	–	–	–	X	X	–	–	–	–
Speech therapist	–	–	–	X	–	–	X	X	–	X
Special educator	–	–	–	–	–	–	X	X	–	X
Specialized nurse	–	–	–	–	–	X	–	–	–	X
Other:	neurologist	–	–	–	–	–	–	–	–	MD; OT
*Diagnostic elements for BCE diagnosis*										
ADI-R	X	X	X	188	X	–	X	–		X
ADOS	X	X	X	135	X	X	X	–		X
Developmental history	X	X	–	–	–	X	–	X^2^		–
Cognitive functioning	X	X	X	135	X	X	X	–		X
Language	–	X	–	43	–	X	X	X		–
Adaptive functioning	Vineland-II	–	Vineland	Vineland–II; N = 43	Vineland	Vineland	Vineland	Vineland–II		–
Observation	Preschool	Preschool; P–C ia	–	Child; N = 53	–	Preschool; Home; P–C ia	Preschool	Child		PC–ERA
Psychiatric assessment	–	X	–	–	X	–	–	–		–
Questionnaires	SCQ, CBCL	CBCL, TRF	–	–	–	–	CBCL; TRF; ADHD RS	–		–
*Other*						Physical and neurological examination	Physical and neurological examination	Psychomotor development		ITSEA; neurological examination

X = specified characteristic applies to all cases of the site; numbers in columns refer to the number of cases the specified characteristic applies to

*ADHD RS* ADHD Rating Scale (DuPaul et al.^1998^), *CBCL* Child Behavior Checklist (Achenbach and Rescorla 2000), *Dev pr* developmental problems, *ESAT* Early Screening of Autistic Traits Questionnaire (Dietz et al. 2006), *GP* general population, *HR* high-risk population, *ITSEA* Infant-Toddler Social and Emotional Assessment (Carter et al. 2003), *MD* medical doctor, *OT* occupational therapist, *PC*-*ERA* Parent–Child Early Relational Assessment (Clark 1995), *P*–*C ia* parent–child interaction, *SCQ* Social Communication Questionnaire (Rutter et al. 2003a), *TRF* Teacher Report Form (Achenbach and Rescorla 2000), *Vineland* (Sparrow et al. 1984), *Vineland*-*II* (Sparrow et al. 2005)

^a^87 % with positive score on ESAT, 13 % with negative score on ESAT but with clinical concern

^b^Most often ADI-R

The sites included children from various backgrounds: some samples were based on diagnostic assessment of toddlers/children considered ‘at risk’ of ASD following screening in general or high risk populations (NL Nijmegen, part of the UK, Spain, Finland), whereas others were based on diagnostic assessment of clinical referrals for ASD or other developmental problems based on parental and/or professional concern (Sweden, NL Utrecht, Iceland, Macedonia, France). The children from Israel were included for research into the relationship between use of medication by mothers during pregnancy and social communicative development and temperament of their children after birth. They were not considered at risk for ASD for research or clinically and were recruited from the general population, however, a large proportion was born prematurely. These children were included for determining sensitivity and specificity.

Additionally, as shown in Table 2, not all of the sites had data in all three developmental cells, or the numbers were too small for reliable and valid analyses with the revised algorithms. The sample of N = 7 in the 12-20/NV21-47 TD cell is very small even in the total sample. Also, the composition of the data differed over sites. For example, while most sites included children with ASD as the majority (over 58 % in eight out of 10 sites, with five over two-thirds), in the other two subsamples TD was dominant. Another example is that the Finnish sample contained children who were clinically referred for concerns on ASD based on population screening, but who were not diagnosed with ASD (yet) after a thorough diagnostic procedure. At the same time, the sample from Israel contained children who were *not* specifically at risk for ASD.

#### Best Clinical Estimate Diagnosis (BCE)

For all toddlers and young preschoolers, a clinical diagnosis was based on thorough diagnostic procedures in expert teams including at least a child psychologist and/or child psychiatrist (see Table 3 for specific procedures and disciplines).

#### Autism Diagnostic Interview-Revised (ADI-R)

All toddlers and young preschoolers in the study had been administered an ADI-R, by a trained psychologist, psychiatrist or speech and language pathologist with research reliability in administration and scoring of the interview. Most often the standard ADI-R was administered and in 249 cases (Israel and UK CHAT study) the toddler ADI-R was administered. In Sweden, the Netherlands, Finland, Spain, France and Israel, an officially translated, approved and published ADI-R was available. In Iceland and Macedonia a translated and approved version of the ADI-R was available although this had not been published.

The mean ADI-R domain scores (Table 2) varied over the sites. These scores did not seem to be systematically related to recruitment method. For example, the first two samples differed in background, yet had relatively comparable mean domain scores. Compared to the US samples, in the current sample, ASD children had relatively low scores on the SA/SC domain, especially in the PH21-47 cell. Additionally, the NS children from the current sample seemed to have relatively high scores on the SA/SC domain. Furthermore, all RRB scores seemed to be relatively low. However, the differences between the current and the US samples could not be formally tested, since the original datasets of the US samples would have been needed for that.

#### Non-verbal Level of Functioning

Level of nonverbal cognitive functioning was available for 983 cases (89 %), most often measured with the Mullen Scales of Early Learning (MSEL; Mullen 1995), the Merrill-Palmer–Revised Scales of Development (Roid and Sampers 2005), or the PEP-R (Schopler et al. 1990). For the Mullen, NVIQ was based on fine motor (FM) and visual reception (VR) age equivalents: NVIQ = (mean age equivalent on FM and VR/chronological age in months) × 100. For the Merrill-Palmer, NVIQ was calculated as (mean age equivalent on cognitive and fine motor/chronological age in months) * 100. For the PEP-R, NVIQ was calculated as: (mean developmental age in months on all subscales except for the verbal scale/chronological age in months) * 100. The mean NVIQ differs over the sites, ranging from 40.4 to 113. This is important, since the level of NVIQ might have influenced scores on the ADI-R if these were correlated in the current sample. In that case, the differences in NVIQ might explain the differences in mean domain scores on the ADI-R. Pearson r correlations seemed to indicate that the domain scores were slightly more related to NVIQ in the UK and Spain samples than in the Sweden and NL Nijmegen samples (Sweden: -.00 through -.30; NL Nijmegen: -.10 through -.29; UK: -.19 through -.65; Spain: -.17 through -.58). Macedonia had the highest domain scores and the lowest NVIQ, however, the n was too small for Pearson r correlation (5 in SW, 15 in PH cell).

### Design and Analyses

The current sample was divided into the three developmental cells (12-20/NV21-47; SW21-47 and PH21-47) as described by Kim and Lord (2012). Revised algorithm scores and classifications were calculated for each case as applicable with respect to developmental cell. For all analyses, ADI-R item scores of 3 were transformed into 2.

Several analyses were performed in order to investigate the ADI-R algorithms for toddlers and preschoolers. First, we investigated the goodness of fit of the three factor structure of the revised ADI-R algorithms, based on the items they contain, with exactly the same Mplus (Muthén and Muthén 2007) Confirmatory Factor Analysis model for categorical data as applied in the algorithm development study (2012) and the replication studies (2013). This was applied to the whole sample, including ASD, NS and TD. Second, also including all diagnostic groups, correlations between the algorithm scores and participant characteristics were calculated in order to examine how independent the algorithm scores were from NVIQ and age. Third, sensitivity and specificity of the algorithms were calculated for the distinction between ASD and NS (without TD), and outcomes were compared to the former studies. This was done for the research criteria and the clinical criteria separately. Kim and Lord (2012) created the two sets of criteria in order to be able to decide which would be most appropriate for a specific setting. The clinical cutoffs aim for maximum sensitivity with adequate specificity, whereas the research cutoffs aim for maximum specificity with adequate sensitivity. Depending on whether the ADI-R is used to include possible cases, or definite cases, a clinician can choose which cutoff to apply. For some research aims it may be important to include definite cases only, for example when time consuming and expensive research is conducted. On the other hand, researchers investigating the broader spectrum may want to include a group with milder symptoms as well. With Receiver Operating Characteristic (ROC) analyses, the effect of including the IGP domains in the total scores in the 12-20/NV21-47 and SW21-47 cells, and of omitting the RPI score for the PH21-47 cell was examined on the balance between sensitivity and specificity. For these analyses, following the study of Kim and Lord (2012), TD was excluded in order to prevent artificial increase of the sensitivity and specificity. Fourth, the applicability of the ranges of concern proposed by Kim and Lord (2012) was investigated in the current sample. Therefore, we compared the percentages of children with a clinical ASD, NS or TD diagnosis within each range to those in each range in the former studies. Last, in order to investigate the predictive value of the revised algorithm domains, logistic regressions were performed in the sample with a clinical ASD or NS diagnosis. TD was not included in this comparison due to comparability with the former studies. Additionally, comparing TD and ASD does not resemble clinical practice. Due to the diagnostic group differences on age and NVIQ (see Table 1), logistic regression analyses were applied with age and NVIQ in the analyses, comparable to the CPEA/STAART study (Kim et al. 2013).

## Results

### Confirmatory Factor Analyses

Table 4 shows the proposed three factor solution of the revised algorithm in the current sample. This solution had satisfactory indices of goodness of fit in all developmental cells: Comparative Fit Indices (CFI) ranged from .889 to .929 (CFI between .9 and 1.0 indicates a good fit of the proposed model) and the Root Mean Square Error Approximations (RMSEA) ranged from .055 to .063 (RMSEA below.08 indicates a satisfactory goodness of fit). Correlations between factors were .68–.90 for the 12-20/NV21-47 cell, .64–.92 for the SW21-47 cell and .67–.83 for the PH21-47 cell. In all cells, correlations between the SA/SC factor and the IGP/RPI factor were the highest.

**Table 4:** New ADI-R algorithm loadings in non-US sample

12-20/NV21-47	Factor loadings	SW21-47	Factor loadings	PH21-47	Factor loadings
*Social affect*		*Social affect*		*Social affect*	
C. Attention to voice	.82	C. Attention to voice	.70	C. Attention to voice	.69
C. Direct Gaze	.80	C. Direct Gaze	.79	C. Direct Gaze	.80
C. Social Smiling	.77	C. Social Smiling	.75	C. Nodding to mean yes	.69
C. Seeking to share enjoyment	.68	C. Seeking to share enjoyment	.65	C. Seeking to share enjoyment	.78
C. Range of facial expression	.80	C. Range of facial expression	.79	C. Range of facial expression	.78
C. Inappropriate facial expression	.56	C. Inappropriate facial expression	.63	C. Offers comfort	.72
C. Appropriateness of social response	.81	C. Appropriateness of social response	.73	C. Pointing	.76
C. Interest in children	.85	C. Interest in children	.71	C. Directing attention	.80
C. Response to approaches of children	.81	C. Response to approaches of children	.75	C. Quality of social overtures	.82
		C. Quality of social overtures	.77	C. Social chat	.79
				C. Use of other’s body	.54
*Repetitive and restricted behaviors*		*Repetitive and restricted behaviors*		*Repetitive and restricted behaviors*	
E. Repetitive use of objects	.81	E. Repetitive use of objects	.89	C. Stereotyped language	.87
E. Hand mannerisms	.65	E. Hand mannerisms	.74	E. Hand mannerisms	.74
E. Complex mannerisms	.79	E. Complex mannerisms	.75	E. Complex mannerisms	.68
E. Unusual sensory interests	.75	E. Unusual sensory interests	.73	E. Unusual sensory interests	.56
		E. Unusual preoccupations	.55	E. Unusual preoccupations	.31
		E. Compulsions/rituals	.51	E. Compulsions/rituals	.42
*Imitation, gestures and play*		*Imitation, gestures and play*		*Reciprocal and peer interaction*	
C. Pointing	.81	C. Pointing	.80	C. Appropriateness of social response	.77
C. Gestures	.86	C. Gestures	.80	C. Interest in children	.83
C. Imitation of actions	.82	C. Imitation of actions	.76	C. Response to approaches of children	.92
C. Offering to share	.79	C. Offering to share	.67		
C. Imaginative play	.83	C. Imaginative play	.79		
C. Directing attention	.90				
*CFI:* .929 (.952^a^, .948^b^, .852^c^)		*CFI:* .889 (.943^a^, .908^b^, .892^c^)		*CFI:* .912 (.960^a^,.913^b^, .806^c^)	
*RMSEA:* .060 (.069^a^, .057^b^, .084^c^)		*RMSEA:* .063 (.062^a^, .077^b^, .066^c^)		*RMSEA:* .055 (.053^a^, .053^b^, .093^c^).	

^a^Values from Kim and Lord (2012)

^b^Values from Kim et al. (2013), CPEA sample

^c^Values from Kim et al. (2013), NIMH sample

### Correlations with Participant Characteristics

Correlations of ADI-R algorithm domain scores with age did not exceed an *r* of .4 in any of the developmental cells. With NVIQ, correlations did not exceed an *r* of .5. Excluding children with TD from the analyses lead to slightly lower correlations (*r* < .4 for age and NVIQ).

### Sensitivity and Specificity

Sensitivity and specificity could only be calculated for ASD versus non-spectrum, since no differentiation between autistic disorder (AD) and non-autism ASD (e.g. pervasive developmental disorder—not otherwise specified) had been made within the ASD group. The outcomes are presented in Table 5.

**Table 5:** Sensitivity and specificity of cutoff criteria ADI-R Toddler algorithms for ASD versus non-spectrum

	Sensitivity non-US sample (95 % CI)	Sensitivity US samples	Specificity non-US sample (95 % CI)	Specificity US samples
*12*-*20/NV21*-*47*				
Research cutoff = 13	.66 (.60–.72)	.77^a^/.76^b^/.85^c^	.95 (.86–.99)	.85^a^/.94^b^/.64^c^
Clinical cutoff = 11	.78 (.72–.82)	.85^a^/.85^b^/.90^c^	.93 (.84–.98)	.70^a^/.94^b^/.64^c^
*SW21*-*47*				
Research cutoff = 13	.53 (.46–.60)	.71^a^/.72^b^/.89^c^	.89 (.81–.95)	.90^a^/.92^b^/.89^c^
Clinical cutoff = 8	.80 (.73–.85)	.94^a^/.96^b^/.97^c^	.70 (.59–.79)	.81^a^/.83^b^/.58^c^
*PH21*-*47*				
Research cutoff = 16	.45 (.37–.53)	.70^a^/.80^b^/.67^c^	.93 (.86–.97)	.82^a^/.94^b^/.86^c^
Clinical cutoff = 13	.56 (.48–.64)	.80^a^/.89^b^/.89^c^	.81 (.73–.87)	.70^a^/.94^b^/.76^c^

^a^Values from Kim and Lord (2012), Michigan sample

^b^Values from Kim et al. (2013), CPEA sample

^c^Values from Kim et al. (2013), NIMH sample

In the 12-20/NV21-47 cell, specificity for ASD was high, .93 for the clinical and .95 for the research algorithm cutoff. Sensitivity in this cell was .78 for the clinical and .66 for the research cutoff.

In the SW21-47 cell, the clinical cutoff was associated with a specificity of .70, with a sensitivity of .80, and the research cutoff resulted in a higher specificity (.89) with a low sensitivity of .53. In the PH21-47 cell, the specificity was again highest for the research criteria (.93) with a sensitivity of .45 only, and lower for the clinical criteria (.81), with a sensitivity of .56.

Further investigation of the separate sensitivities was undertaken for those sites with a sample size of over a hundred cases and enough children with ASD and NS (Sweden; NL Nijmegen; UK; and Spain). The large majority of the data from Israel represented TD, therefore, sensitivity and specificity were not calculated for this sample. Sensitivities varied over the sites: in the Netherlands and Sweden .31–.47 for research cutoffs and .47–.71 for clinical cutoffs; in the UK and Spain .64–.91 for research cutoffs and .64–.98 for clinical cutoffs.

Based on the ROC analyses, the Areas under the Curve (AuC) indicated that the algorithms as proposed by Kim and Lord (2012) were valid when comparing a clinical diagnosis of ASD versus non-spectrum [AuC.93 (95 % CI .90–.97) for 12-20/NV21-47; AuC.83 (95 % CI .78–.88) for SW21-47; AuC.77 (95 % CI .71–.82) for PH21-47]. These analyses investigated a continuous measure of criterion related validity, based on the total scores of two or three domains (the total scores on the proposed algorithms in each cell). Note that the domains were not examined separately. Experimentally adding the IGP domain items to the total score for the 12-20/NV21-47 and SW21-47 cell resulted in an AuC that resembled the one based on the two domain total score [.94 (95 % CI .90–.97) for 12-20/NV21-47;.84 (95 % CI .79–.89) for SW21-47]. Excluding the RPI domain items from the total score for the PH21-47 cell also resulted in a comparable AuC (.78; 95 % CI .73–.84). Adding or omitting the IGP/RPI domain items thus did not seem to affect the sensitivity or specificity *over the range of total scores* on two or three domains combined in the current sample.

### Ranges of Concern

The ranges of concern as defined by Kim and Lord (2012), aiming for 80 % of the children with ASD in the ranges of mild-to-moderate or moderate-to-severe concern and 95 % of the TD children in the little-to-no concern range, seemed more or less applicable to the 12-20/NV21-47 and SW21-47 developmental cells in the current sample: In the 12-20/NV21-47 cell, 77.2 % of the 246 children with ASD fell into the ranges of mild-to-moderate or moderate-to-severe concern and in the SW21-47 cell 79.7 %. Of the TD children 90.5 % in the SW21-47 cell fell into the no-to-little concern range. In the 12-20/NV21-47 there were only 7 children in the TD group, therefore the number in this cell is too small to analyze reliably. Of the NS children, 6.6 % in the 12-20/NV21-47 and 30 % in the SW21-47 cell fell into the risk ranges, percentages that fell within the ranges in the Michigan sample (30–33 %; 2012) and CPEA/STAART sample (6–16 %; 2013). For the PH21-47 cell the results were somewhat different. Whereas 98.2 % of the TD group and 80.8 % of the NS children fell into the little-to-no concern range, only 56.1 % of the children with ASD fell into one of the risk ranges. This means that 43.9 % of children diagnosed with an ASD in the current sample fell into the little-to-no concern range, with total scores of 12 or lower on the ADI-R algorithm.

### Logistic Regressions

Logistic regressions could only be performed for children for who NVIQ was available. With logistic regressions, the contribution of the individual domains to a clinical classification of ASD versus NS was investigated, with all other domains, age and NVIQ in the analyses. In the current sample, the SA/SC domains contributed significantly to a clinical diagnosis of ASD versus NS in all developmental cells [12-20/NV21-47 odds ratio (OR) 1.44, 95 % CI 1.20–1.72, *p* < .001; SW21-47 OR 1.26, 95 % CI 1.12–1.42, *p* < .001; PH21-47 OR 1.27, 95 % CI 1.16–1.40, *p* < .001]. The RRB domain did not affect diagnosis in the 12-20/NV21-47 group (OR .95, 95 % CI .69–1.30; *p* = .742) or in the PH21-47 cell (OR 1.11, 95 % CI .95–1.30, *p* = .196), yet it made a significant contribution in the SW21-47 cell (OR 1.19, 95 % CI 1.01–1.41, *p* = .041). In the 12-20/NV21-47 cell, the IGP domain additionally contributed to the clinical diagnosis, with an OR of 1.34 (95 % CI 1.11–1.62; *p* = .002), in the SW21-47 cell IGP contributed too (OR 1.20, 95 % CI 1.02–1.40, *p* = .025) yet in the PH21-47 cell RPI did not add to a diagnosis (OR .91, 95 % CI .73–1.13, *p* = .368). These analyses revealed that all individual domains independently contributed to the identification of children with ASD, yet that their roles varied over cells.

## Discussion

The current paper aims to make a modest contribution to the literature by examining aspects of the validity of the ADI-R algorithms for toddlers and preschoolers (Kim and Lord 2012) in an independent and large non-US sample (N = 1,104). With respect to construct validity, the three factor structure as found by Kim and Lord (2012) fitted the data well. In the current sample, the specific items fitted well into the specific ADI-R toddler and preschooler domains, in line with the values of Kim and Lord and the replication studies (Kim et al. 2013). The fit indices of the three factor model were satisfactory to good, resembling the ones in the US samples and indicating that the new ADI-R algorithm structure can be applied to the non-US data. Correlations between factors were comparable to those in the CPEA/STAART (*r* = .69–.94; Kim et al. 2013) and NIMH samples (*r* = .55–.99; Kim and Lord 2012) indicating the same high correlations between the three factors. In particular the high correlations between SA/SC and IGP/RPI indicated that these domains were not independent from each other.

Another finding that corroborated the construct validity was the relatively low correlation between the algorithm scores and age and level of cognitive functioning. The levels of these correlations were comparable to those in the Michigan study (r < .5, most < .4; Kim and Lord 2012), and in the CPEA/STAART study (r < .4; Kim et al. 2013) and NIMH study (r ≤ .4; Kim et al. 2013). Nevertheless, the correlation between mean domain scores and NVIQ varied over the sites, with relatively higher correlations for the UK and Spain.

The criterion related validity of the algorithm scores as proposed by Kim and Lord (2012) was satisfactorily high. The proposed combination of domains for classification (SA and RRB in 12-20/NV21-47 and SC, RRB and RPI in PH21-47) corresponded with a clinical ASD diagnosis in the current sample. Further investigation of this criterion related validity indicated that the third factor (IGP/RPI) was not a totally separate factor that reflected a crucial behavioral domain for ASDs in the current sample, even though it did contribute to a clinical classification. Adding the IGP factor in the analyses for 12-20/NV21-47 or SW21-47 cells or omitting the RPI factor in the PH21-47 cell did not affect the criterion related validity of the total scores, which is understandable with the high correlation between this domain and SA/SC (*r* = .83–.92 over the developmental cells). This indicates that algorithm scores based on the *total* of two or three domains were equally valid compared to a clinical diagnosis of ASD in the toddlers and preschoolers in the current sample. Kim and Lord (2012) have reported that no third domain was needed for the algorithm cutoffs in the12-20/NV21-47 and SW21-47 cells, but did include three domains for PH21-47. In the current sample the third domain did not seem to add to the criterion related validity for the PH21-47 cell either. If further research in other independent samples replicated this finding, it would potentially add to the comparability of the algorithms over the cells (each consisting of two rather than three domains), and enhance comparability of scores over time within and between children. Overall, the findings on construct validity indicate that the ADI-R toddler algorithms are well applicable to the non-US data, with valid content and factor structure.

With respect to diagnostic validity, the results from the current study were less consistent with the original study. The *specificities* for the clinical and research cutoffs in the current sample resembled the ones in the US studies, except in the SW21-47 cell, which had a lower specificity on the clinical cutoff (.70). However, the *sensitivities* in the current sample were lower for all developmental cells compared to the original Kim and Lord (2012) study and the CPEA/STAART and NIMH studies (Kim et al. 2013). In the 12-20/NV21-47 cell, the 95 % CI’s of sensitivities overlapped between the current and the US samples, therefore no firm conclusion could be drawn regarding the significance of this difference. In the SW21-47 and PH21-47 cells, the sensitivities (both cutoffs) were significantly lower in the current sample than in the Michigan and CPEA/STAART samples as evidenced by the non-overlapping 95 % CI’s. Compared to the NIMH sample, the sensitivity of the research cutoff in the SW21-47 cell was significantly lower in this non-US sample, however again no firm conclusion could be drawn regarding the clinical cutoff, due to overlapping 95 % CI’s. In the PH21-47 cell, the sensitivity of the clinical cutoff was significantly lower in the current sample, yet the 95 % CI’s of the research cutoff overlapped with the NIMH sample, meaning no firm conclusion could be drawn on the significance of this difference.

These findings indicate that although the content and structure of the algorithms were applicable in the current sample, the sensitivity of the ASD classification based on the reported research and clinical cutoff scores was only moderate and lower than in the Kim and Lord studies (Kim and Lord 2012; Kim et al. 2013).

However, investigation of the ranges of concern revealed that, in the 12-20/NV21-47 and SW21-47 cells, the percentages of children with ASD in the mild-to-moderate and moderate-to-severe ranges approached the ones in the US samples. This indicates that children in these cells with a clinical diagnosis of ASD were recognized as in the concern range, and that the majority of children with TD were indeed found to be in the little-to-no concern range. In contrast, in the PH21-47 cell, only 56.1 % of the children clinically diagnosed with ASD fell into the concern ranges, which means that 43.9 % received a score on the ADI-R in the range of little-to-no concern.

One explanation may be that for the PH21-47 cell, only the SC domain contributed significantly to the clinical diagnosis, instead of contributions from each of the domains (SC, RRB and RPI) as reported in the US samples, possibly indicating a shift in what was important for a clinical diagnosis of ASD in the current sample. In the 12-10/NV21-47 cell, the SA and the IGP domain individually contributed, and in the SW21-47 cell all domains affected ASD diagnosis.

The low sensitivity might be accounted for by the nature of the current sample as compared to the US samples (Kim and Lord 2012; Kim et al. 2013). Most likely, the fact that more than half of the children had been recruited after screening (N = 446; NL Nijmegen, part of UK, Spain, Finland) instead of after clinical referral (N = 352; Sweden, NL Utrecht, Iceland, Macedonia, France) will have influenced these results. The children from Israel were not included in these numbers, since they were neither clinically referred, nor recruited after screening, and purposefully included as TD. Thus, around 40 % of the sample were administered the ADI-R as part of diagnostic assessment in a prospective screening study. In the Michigan, CPEA/STAART and NIMH samples, that were primarily clinically referred samples, it might be expected at this earlier age that symptoms were (on average) more severe than in children identified as ‘at risk’ for autism by a screening instrument. This may in part account for the lower sensitivity we found in our sample. Perhaps, parents of children identified through screening may be less aware of some of the behaviors they are asked about during the ADI-R. This may lead to lower scores on the ADI-R, although other sources of information, including direct observation and information from preschool or daycare, may identify behaviors sufficiently suggestive of an ASD diagnosis. In such cases, the ADI-R scores may be below threshold if the parent is apparently unaware of the unusual behaviors. This may apply particularly to parents of very young children.

The percentages of ASD diagnosis per site were higher than the percentages of children with an ADI-R score above clinical or research cutoff. Only in the UK sample, the difference between diagnosis and percentage above cutoffs was small. In other words, in some to relatively many cases, the diagnostic teams had sufficient indication to establish a clinical ASD diagnosis, even though parents did not report severe problems during the ADI-R. This was not only true for parents recruited through screening (e.g. NL Nijmegen), but also for clinically referred children for ASD or other developmental concerns (e.g. Sweden).

Therefore, the nature of recruitment/referral would be unlikely to be the only explanation. Another consideration might be the relatively low proportion of ASD versus NS (excluding TD) in the SW21-47 and PH21-47 cells in the Dutch, Swedish and Spanish samples (for SW21-47 72, 63 and 48 % ASD respectively; for PH 21-47 50, 52 and 46 % ASD) compared to the US-samples (percentage ASD in SW21-47 Michigan sample 81 %, CPEA/STAART 85 % and NIMH 66 %; PH21-47 Michigan sample 63 %, CPEA/STAART 88 % and NIMH 56 %). With the small sample sizes per site, the focus needs to be on the general picture of the combined dataset and not site-by-site variation.

The lower sensitivity of the research criteria in the current sample than in the former studies (Kim and Lord 2012; Kim et al. 2013) might indicate that using the ADI-R in the current sample as the criterion for inclusion for research studies would have led to small numbers of children included. However, with the satisfactory specificity, they would be definite cases of ASD. Thus, as the authors described (Kim and Lord 2012, p. 91) these criteria would be helpful for researchers who need definite cases. However for researchers investigating the broader range of ASDs, the current findings indicate that the ADI-R may not be very sensitive to identify cases of interest. As acknowledged many times before by the authors of the ADI-R, the ADI-R is not equivalent to the diagnosis and is meant to be used as a tool in the diagnostic procedure and it should be combined with information from other sources to result in a BCE. The current findings suggest that if researchers want to include the broader spectrum of ASDs in their research sample, the ADI-R alone should not be used as the only criterion. However, the current findings are probably due to the nature of the samples and in the particular focus of any given research study should drive decisions on inclusion criteria in research samples.

The findings from the current study were only partly consistent with the findings reported in the US studies. The construct validity resembled former findings, the diagnostic validity was less stable (lower sensitivity). This might however be a consequence of recruitment/referral to the current sample and of the relatively low scores on the ADI-R of the children with a clinical ASD diagnosis. Despite this uncertainty, especially for the 12-20/NV21-47 and SW21-47 cells, the ADI-R algorithms for toddlers and preschoolers were likely to be of considerable value in aiding clinicians as they had to make diagnostic decisions in very young children. At all sites in the current study, clinical diagnoses were based on several sources of information (in addition to the ADI-R and often ADOS) and were assigned by an experienced expert, most often in a team. Detailed information obtained using the ADI-R and ADOS(-2) in a standardized diagnostic procedure has been shown to make specific contributions to the clinical decision-making process (see Kim and Lord 2012; Kim et al. 2013; Charman and Gotham 2013).

### Limitations

Although the total sample size of the current study was large, the sample consisted of children from many different sites and is thus not a true replication study, given the different methodologies for assessment and diagnostic procedures, and ascertainment of samples. The sites provided a wide variety of samples recruited for different purposes (clinical referral/screening, first line/second line); in diagnostic groupings (some TD only, others NS only, others mainly ASD); from several populations (prediagnosed/undiagnosed; specialized departments/generic departments); with a range in severity of symptoms, age distribution (very young only versus broader), number of participants and level of cognitive functioning. However, unfortunately the individual sample sizes were too small to allow any additional analysis for any individual sites.

## Conclusion

The current study indicates that the construct validity of the algorithms for toddlers and preschoolers as proposed by Kim and Lord (2012) was applicable in a large, independent, non-US sample. The selected ADI-R items fitted into the proposed domains SA/SC, RRB and IGP/RPI in the non-US sample as well as in the US sample. This indicates that the theoretical concept of the ADI-R in toddlers and young preschoolers seemed to be the same for US and non-US samples. However, in the current sample somewhat lower diagnostic validity was found, with satisfactorily high specificities but only moderate sensitivities. Although children with a clinical ASD diagnosis in the 12-20/NV21-47 and SW21-47 cells were largely recognized as children in the mild-to-moderate or moderate-to-severe concern ranges, nearly half of the children with a clinical ASD diagnosis in the PH21-47 cell fell into the little-to-no concern range.
